# Honoring the Voices of Older Adults: CBPR Approaches to Build Community Guided Initiatives

**DOI:** 10.1093/geroni/igaf122.1284

**Published:** 2025-12-31

**Authors:** Jordan Lewis

## Abstract

Much of the past research conducted with BIPOC communities was conducted under false narratives, or as “helicopter research,” where researchers would enter the community, gather data, and leave the community without ever discussing or consulting with community members. These past unethical practices, with no community involvement, and no reporting to the communities on how the data was used or published have created significant mistrust. Community Based Participatory Research is an approach conducted as an equal partnership between community members, organizational representatives, and researchers. This symposium will offer a panel of presentations highlighting the importance of community-engaged research studies with BIPOC communities. The first presentation focuses on a study with a southern African American community that created a training program for clinicians on how to communicate in a culturally congruent and respectful way with older southern, African Americans with serious illness and at end of life. The second paper discusses the community engaged practices that developed a culturally relevant, community-delivered intervention to improve social connections among Black caregivers. The third presentation focuses on experiences and lessons learned when developing Elder-based research approaches to Indigenous healthy aging research. The final presentation, discusses the importance of building local and national partnerships to address the crucial need of sharing culturally sensitive knowledge related to brain health, resources and support for dementia caregivers, and dementia knowledge in remote Alaska Native communities. The presenters will discuss the partnership building process and its impact on communities on the family, the community, and at the state level. This is a collaborative symposium between the Community-Engaged Research and Generativity and Aging Interest Groups.

